# Optimization of Ultrasound-Assisted Extraction of Bioactive Compounds from *Acacia Seyal* Gum Using Response Surface Methodology and Their Chemical Content Identification by Raman, FTIR, and GC-TOFMS

**DOI:** 10.3390/antiox10101612

**Published:** 2021-10-13

**Authors:** Tahani Maher, Nassereldeen A. Kabbashi, Mohamed E. S. Mirghani, Md Z. Alam, Djabir Daddiouaissa, Ferid Abdulhafiz, Mohd Farhan Hanif Reduan, Jihad I. Omran, Mohammad Khairul Azhar Abdul Razab, Arifullah Mohammed

**Affiliations:** 1 Biotechnology Engineering Department, Kulliyyah of Engineering, International Islamic University Malaysia (IIUM), P.O. Box 10, Gombak, Kuala Lumpur 50728, Malaysia; alawdat.tahani@live.iium.edu.my (T.M.); zahangir@iium.edu.my (M.Z.A.); 2 International Institute for Halal Research and Training (INHART), International Islamic University Malaysia (IIUM), Jalan Gombak, Kuala Lumpur 53100, Malaysia; elwathig@iium.edu.my (M.E.S.M.); daddiouaissa.djabir@live.iium.edu.my (D.D.); 3 Faculty of Agro-Based Industry, Jeli Campus, Universiti Malaysia Kelantan, Jeli 17600, Malaysia; ferid.f18e006f@siswa.umk.edu.my; 4 Department of Paraclinical Science, Faculty of Veterinary Medicine, Universiti Malaysia Kelantan, Pengkalan Chepa, Kota Bharu 16100, Malaysia; farhan.h@umk.edu.my; 5 Biomedical Science Programme, Faculty of Health Sciences, Universiti Kebangsaan Malaysia, Jalan Raja Muda Abdul Aziz, Kuala Lumpur 50300, Malaysia; p94173@siswa.ukm.edu.my; 6 School of Health Sciences, Health Campus, Universiti Sains Malaysia, Kubang Kerian 16150, Malaysia

**Keywords:** *Acacia Seyal* gum, gum Arabic, ultrasound-assisted extraction, response surface methodology, Raman spectroscopy, FTIR spectra, GC-TOFMS

## Abstract

*Acacia Seyal* gum (ASG), also known as gum Arabic, is an antioxidant-rich soluble fiber. ASG has been reported to have many biological activities, including anticancer, antidiabetic, antiulcer, and immunomodulatory activity. Extraction of bioactive compounds from ASG is commonly performed using conventional extraction methods. However, these techniques have certain limitation in terms of extraction time, energy, and solvent requirements. Ultrasound-assisted extraction (UAE) could be used as an alternative technique to extract bioactive compounds in less time, at low temperature, and with less energy and solvent requirements. In this study, the UAE extraction of ASG was optimized using response surface methodology (RSM). A face-centered central composite design (FCCCD) was used to monitor the effect of different independent factors of ultrasound operation (sonication time, temperature, and solvent ratio) on ASG extraction yield. In addition, screening and characterization of phytochemicals in 60% ethanol ASG extract was carried out using Raman microscopy, Fourier transform infrared spectroscopy (FTIR), and gas chromatography time-of-flight mass spectroscopy (GC-TOFMS) analysis. The results indicated that, under optimal conditions (extraction time 45 min, extraction temperature 40 °C, and solid–liquid ratio of 1:25 g/mL), the yield of ASG was 75.87% ± 0.10. This yield was reasonably close to the predicted yield of 75.39% suggested by the design of experiment. The ANOVA revealed that the model was highly significant due to the low probability value (*p* < 0.0001). Raman spectrum fingerprint detected polysaccharides, such as galactose and glucose, and protein like lysine and proline, while FTIR spectrum revealed the presence of functional groups peaks value of alkanes, aldehydes, aliphatic amines, and phenol. GC-TOFMS spectroscopic detected the presence of strong d-galactopyranose, carotenoid, and lycopene antioxidant compounds. In conclusion, this study demonstrated that the UAE technique is an efficient method to achieve a high yield of ASG extracts. The selected model is adequate to optimize the extraction of several chemical compounds reported in this study.

## 1. Introduction

Over the last few years, the use of natural compounds for the prevention and treatment of various diseases is becoming increasingly popular and they have shown significant results in the treatment of cancer, Alzheimer, metabolic disorders, inflammations, arthritis, etc. [1]. Beside its therapeutic values, the utilization of natural compounds from various sources has been increased in the food and pharmaceutical industries [2]. As a source of natural health-benefitting compounds, ASG has been used in folk medicine globally. ASG is a dried secretion from trees of *Acacia Senegal* and *Acacia Seyal*. These trees are primarily grown in sub-Saharan African countries, particularly Sudan, Chad, Uganda, and Eritrea. ASG provides a rich source of antioxidant and is reported to have many biological activities including anticancer, anti-inflammatory, hypoglycemic, antidiabetic, antioxidant, and antiulcer activity [3,4]. The biological activity of ASG is associated with its high flavonoid and polyphenolic components [5,6]. Furthermore, it is reported to have abundant terpenoids, tannins, lignans, alkaloids, coumarins, and quinones [7,8].

Drug development is highly dependent on the natural ingredients derived from medicinal plants. However, these substances are usually found in plants in a small quantity. Therefore, developing an effective and selective techniques for extracting and isolating such bioactive natural compounds is greatly important.

Conventional extraction procedures, such as percolation, maceration, and reflux, often utilize organic solvents and need a significant volume of solvents and a long extraction period. Previous studies demonstrate that the conventional methods employed for extracting bioactive compounds from ASG are time consuming and have low yields with low amounts of bioactive compound [3,9].

To overcome these problems, novel and environment-friendly (green chemistry) extraction technologies have been suggested including pressurized liquid extraction (PLE), ultrasound-assisted extraction (UAE), microwave-assisted extraction (MAE), and supercritical fluid extraction (SFC) [10]. UAE is simpler and faster than other extraction methods. UAE consider a sustainable and economically feasible technology suitable for the extraction heat-sensitive compounds. Moreover, it reduces energy usage while producing safe and greater yield of product [11,12]. UAE is appropriate for the extraction of thermolabile and unstable compounds as well as the extraction of a variety of natural plant metabolites [11].

UAE extraction efficiency is affected by the properties of the extraction solvent, the particle size of the raw materials, the solvent-to-solid ratio, the extraction temperature, and the extraction time. Hence, to achieve maximum extraction yield, optimization of extraction techniques by response surface analysis is required to study the influence of different independent variables such as solid–liquid ratio, extraction temperature, extraction time. RSM is an effective optimizing method that requires a reduced number of experimental runs to assess multiple parameters and their interactions [13,14].

Medicinal plants are well known to have chemically diverse compounds produced in very low quantities and are prone to variation due to environmental and other factors [15]. As the use of medicinal plants or natural products continues to gain huge popularity globally, there is a dire need for the detail biochemical-content studies [16,17]. Therefore, advanced analytical techniques are appropriate for the separation, identification, and quantification of compounds. Several advanced analytical technologies have been used in recent years, including Raman spectroscopy, Fourier transform infrared spectroscopy (FTIR), and gas chromatography time-of-flight mass spectroscopy (GC-TOFMS). Raman spectroscopy is increasingly being used to examine medicinal plants to identify and detect natural organic materials and efficiently and precisely identify the chemical components of herbal medications [18]. Raman spectroscopy uses benefits from its non-destructive nature. It is a non-invasive method and can be applied to any sample’s physical state with no sample size restriction [19]. The FTIR technique is used to detect the presence and characterize unknown functional groups present in a plant sample [20]. On the other hand, GC-TOFMS is used to detect the occurrence of bioactive compounds quantitatively and qualitatively. It also detects compounds that are present at very low concentrations and expands the opportunity to explore new bioactive compounds [21].

In this study, 60% ethanol was used as the extraction solvent based on preliminary study results that showed that 60% ethanol produced a high extraction yield. In addition, the polysaccharides extracted from ASG using this ethanol concentration showed the highest bioactivity. Hence, we aim to extract compounds with useful biological activity from ASG to be used later for further research. The objective of this study was to employ response surface methodology to investigate the effect of different UAE extraction process variables (extraction temperature, ultrasonic extraction time, and solid–solvent ratio) on yields of extracts. In addition, the phytochemical compounds present in 60% ethanol extract were identified using Raman spectra, FTIR, and GC-TOFMS techniques.

## 2. Materials and Methods

### 2.1. Raw Material and Reagents

ASG exudate was purchased from the local farmers market in El-Obeid, State of North Kurdufan, Sudan. The collection of gum by the farmers took place during the dry season from November to May 2017. The sample was then verified by experts from the Sudanese Ministry of Forestry and Agriculture. The impurities and pieces of bark were removed by hand and then the gum was crushed into fine powder by using mortar and pestle and then sieved using Fisher brand USA standard testing sieve (Fisher Scientific, Waltham, MA, USA) with 1.40 mm mesh size. The final ASG powder was packed in polyethylene zipped bags (12 cm × 12 cm) and stored at 4 °C until further analysis. Sampling was performed only one time. Ethanol used in the extraction was of analytical grades and purchased from HmbG chemical (HmbG GmbH, Hamburg, Germany).

### 2.2. Plant Sample Extraction

Extractions were performed following the method of Adwan et al. [1] and Esmaeili et al. [22] with some modification using an ultrasonic bath, model DSA100-SK2 (Fuzhou Desen Precision Instruments Co., Ltd., Fujian, China). The UAE experiments were carried out at different ranges of solid-to-liquid ratios (1:15–1:25 g/mL), sonication temperatures (30–50 °C), and sonication times (30–60 min) following the designated experimental design.

The gum powder (3 g) was dissolved in 30 mL of 60% ethanol (*v*/*v*) (ethanol was diluted with distilled water), and then it was covered with aluminum foil and extracted with a 40 kHz sonication power in an ultrasonic bath under the specified conditions. Preliminary investigations and other relevant research were used to identify the independent variables and their fluctuation ranges [23]. The experiments were carried out in which the time, temperature, and solid-to-liquid ratios were chosen as per the values suggested by the RSM design, and the extracts were filtered and centrifuged at 15,000× *g* for 10 min. The solvent was discarded using a rotary vacuum evaporator (Büchi rotavapor, Buchi, Flawil, Switzerland) at 40 °C, followed by freeze drying for 72 h in a freeze dryer (Scanvac Cool Safe 55-4, Scanvac, Denmark) The extraction yield value of ASG was calculated after each experiment. The dried extracts were kept at −20 °C for further analysis [12].

### 2.3. Ultrasound-Assisted Extraction (UAE) Optimization

The design of the experiment was applied to evaluate and observe the impacts of extraction parameters, extraction method on ASG extraction yield (%), and the chemical content of bioactive compounds obtained. The preliminary experiments showed several factors affecting ASG extraction in addition to the type of the extracting solvent, such as the extraction temperature, time, and solid–liquid ratio [24]. All the experiments were performed in triplicates and the results expressed as mean values ± standard error. The extraction yield (%) was defined as the response (Y).

### 2.4. Experimental Design

The RSM was used to design the experiments based on the face-centered central composite design (FCCCD), which included both axial and central points. The total number of samples examined under RSM were 20 (20 experimental runs), and each run was subjected to triplicate analysis. The optimal conditions were determined using analysis of variance (ANOVA), contour plots, and residual plots. Ultrasonication time, temperature, and solid–liquid ratio were independent factors tested in 20 experimental runs to get the optimal conditions for UAE of ASG. The yield was chosen as the design experiment’s response (Y). For the factor assessments, the independent variables were adjusted to ranges between –1 and +1. The variables’ levels in the RSM design were studied at two levels, low (−1) and high (+1), as shown in Table 1. The RSM statistical analysis was employed using Design Expert Software v12.0.0 (Stat-Ease Inc., Minneapolis, MN, USA).

The parameters employed were extraction time (30–60 min), extraction temperature (30–50 °C), and solid–liquid ratio (15–25). The extraction yield of gum was calculated using the final dry weight of extracted gum relative to the original gum powder [25]. Equation (1) was used to determine the extraction yield:

(1) $$Extraction\;yield\;(\%)=\frac{\mathrm{Weight}\;\mathrm{of}\;\mathrm{the}\;\mathrm{extract}\;\mathrm{after}\;\mathrm{extraction}\;\left(g\right)}{\mathrm{Weight}\;\mathrm{of}\;\mathrm{the}\;\mathrm{original}\;\mathrm{sample}\;\left(g\right)\;}\;\times \;100$$

Several studies confirmed the influence of the extraction method and type of solvent on the extract yield, phytochemical content, and biological activity [11,23]. This study used the phytochemical screening and chemical characterization of secondary metabolites in ASG extract using ultrasound-assisted extraction with 60% ethanol to explore the bioactive metabolites and their structures present in the sample.

### 2.5. Raman Spectroscopy Spectra

The ASG-optimized powder was assessed by Raman analysis in the INHART laboratory, International Islamic University Malaysia, Jalan Gombak, 53100, Selangor, Malaysia using a Renishaw System-1000 spectrometer from Wotton-Under-Edge, UK, paired with a diode laser at a wavelength of 785 nm and a 50 mW output power as an excitation source. Using a series of neutral density filters, the laser intensity on the sample may be adjusted up to around 5 mW. While considering possible sample inhomogeneity, a 50× objective lens was used to concentrate the light on the sample. The spectrum was captured at a magnification of 20×, with an extended Raman range of 100–3200 cm^−1^, an exposure time of 10 s, and laser power of 1% 785 nm edge. The spectrum was processed with the computer peak acquisition and Microsoft Excel software.

### 2.6. FTIR Spectroscopy

Spectra were measured with an FTIR spectrometer (Nexus 670 Fourier transform infrared spectrometer, (Thermo Scientific, Waltham, MA, USA) with a PC-based software-controlled instrument for operation and data processing. The powdered samples were put onto the tiny crystal area after it was cleaned, and the pressure arm was placed over it. The spectrum was obtained after applying force to the sample and pressing it onto the diamond surface. Spectra were recorded at 400–4000 cm^−1^ scanning range and 1 cm^−1^ resolution. The average spectra were evaluated. For comparison between different spectra, multiple-point baseline correction and spectra normalization were done. The samples were examined in triplicates. The spectral data were compared to the previous research studies on FTIR of various chemical compounds to identify the functional groups that present in the sample.

### 2.7. Gas Chromatography Time-Of-Flight Mass Spectrometry (GC-TOF-MS) Analysis

The required quantity of ethanolic ASG crude extract was filtered using a nylon syringe filter (0.45 µm). In the split mode (10:1), 2 µL of plant extract was administered before being subjected to GC-TOFMS analysis. The analysis was performed by using Thermo GC ultra Clarus 500 system and gas chromatograph integrated to a mass spectrometer (GC-TOFMS) supplied with an Elite-I-fused RMS 5 silica capillary column composed of 100% Dimethylpolysiloxane as described by Daddiouaissa et al. [26]. As a carrier, helium gas was utilized at a constant flow rate of 1 mL/min, with a sample injection volume of 1 μL and a sample split ratio of 10:1. The injector and ion source temperatures were set at 250 °C and 260 °C, respectively. The oven temperature was set to rise from 110 °C to 260 °C at a rate of 5 °C/min, with a 3 min isothermal ending at 260 °C. An electron ionization device with a 70 eV ionizing energy was utilized to detect mass spectra, with a mass scanning range of 10–400 m/z. For handling mass spectra and chromatograms, Turbo mass software was used. The GC analysis was done in triplicate.

#### Component Identification

The National Institute of Standards and Technology (NIST) database was used to identify components and interpret the mass spectrum of GC-TOFMS (https://www.nist.gov/srd/nist-standard-reference-database-1a-v14) (Accessed on 1 September 2021). Based on the spectra recorded in the NIST and Wiley libraries, unknown components were compared to known components.

### 2.8. Statistical Analysis

RSM statistical analysis was employed using Design Expert Software v12.0.0 (Stat-Ease Inc., Minneapolis, MN, USA). All experimental results obtained were expressed as means ± SD, data were analyzed by analysis of variance (*p* < 0.05), and the means were separated by Duncan’s multiple range tests. All analyses were performed in triplicates.

## 3. Results and Discussion

### 3.1. Optimization of Acacia Seyal Gum Extraction Parameters by RSM

The FCCCD under RSM was employed to investigate the optimal parameters of the three variables (extraction time, extraction temperature, and solid–liquid ratio) to maximize the extraction yield. The experimental results and the predicted yield obtained from the regression equations are presented in Table 2. The highest order polynomials were used to choose the models. The resulting data were fitted with the quadratic model as suggested by the software, and each experimental run showed the interaction between each factor level where the response was the extraction yield (%).

ASG had a wide range of yields from 64% to 76%, and the maximum yield was found at the extraction time of 45 min, an extraction temperature of 40 °C, and the solid–liquid ratio of 25 g/mL. The conditions of the optimal process were analyzed to obtain high extraction yields. These results demonstrated that the yield (%) was maximal with the mid value of extraction temperature (B), mid values of extraction time (A)m and high values of solid–liquid ratio (C). On the other hand, it was found to be minimal at the high value of extraction time (A), high values of extraction temperature (B), and low values of solid-to-liquid ratio (C). The importance of A and B were found to significantly affect the extraction efficiency (Table 3).

### 3.2. Statistical Data Analysis

Based on a 95% confidence interval, the analysis of variance was used to verify the significant impacts of process variables on the ASG yield. The ANOVA results show that the interactive parameters A, B, A^2^, and B^2^ were the significant parameters, while the linear coefficient C and the interactions terms AB, AC, and BC, as well as the quadratic coefficient C^2^ were insignificant (Table 3). However, these interaction terms were taken into consideration as they maintain the current model. In addition, the lack of fit (0.38) is considered not significant (*p* > 0.05), which means that the model fits well.

The ANOVA model analysis for the RSM gave the quadratic results shown in Table 3. According to the resulting analyses, the impact of each variable on the experimental response showed good prediction, where the predicted R² of 0.89 is closer to the adjusted R² of 0.96 with a difference of less than 0.2. Thus, the model is better fitted when the coefficient of determination R^2^ value is closer to one. As a result, R^2^ values showed that the regression model for each response accurately represented the system’s actual behavior. Based on regression coefficient values, extraction time A and extraction temperature B showed a positive effect, and the positive regression coefficient indicates that increasing the extraction time would improve the ASG extraction yield. The negative regression coefficient for the solid–liquid ratio means that its increase would decrease the ASG extraction yield. The AB and B regression coefficients were also positive. On the other hand, a low coefficient of variance (CV%) of 1.35 for all the responses showed an excellent accuracy and reliability of the model. Commonly, if its CV is less than 10%, the model is considered reasonably reproducible [27]. Adequate precision measures the signal-to-noise ratio, where values that are more than 4 are considered desirable. The adequate precision of 18.26 of the extraction yields indicates an adequate signal, and the model generated can be used to navigate the design space.

The model equations for the response shows the relationship between extraction time, temperature, and solid–liquid ratio and yield (Equation (2)).

(2) $$Y=-41.4910\;+\;2.2271\times A\;+\;3.0713\times B\;+\;0.2716\times C\;+\;0.00389\times A\times B\;+\;0.00221\times \;A\times C\;+\;0.001675\times B\times C-{0.00268\times A}^{2}-{0.0403\times B}^{2}-{0.08\times C}^{2}$$

where Y is the predicted responses (yield %), and A, B, and C are the coded values for the independent variables, in which A is time, B is temperature, and C is the solid–liquid ratio, respectively.

The experimental error with a 95% confidence interval (CI) for the means was determined based on the differences between observed and predicted responses in order to evaluate the variable effects and their interactions (Table 4).

RSM was used to create various forms of 3D response surface contour plots to show the anticipated model equation and, therefore, the interaction between the different factors and response. These graphs demonstrated the effect of extraction time, solid–liquid ratio, and temperature on the percentage of the extraction yield.

### 3.3. The Interaction Response Effects

**Figure 1:**
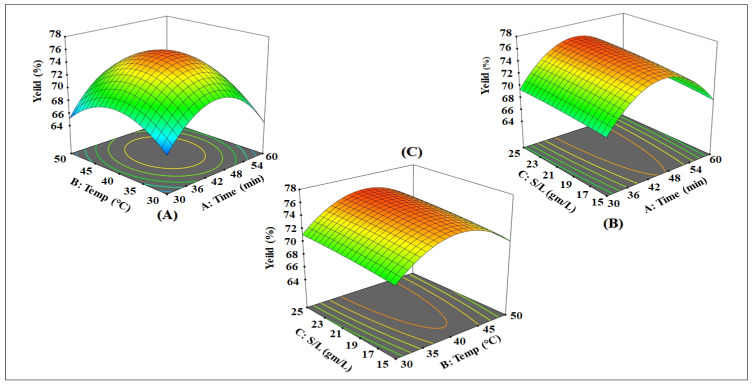
3D plots representing the effects of (**A**) extraction time and temperature, (**B**) time and solid–liquid ratio, and (**C**) extraction temperature and solid–liquid ratio.

As can be observed, the optimal conditions were determined by maximizing the desirability of the responses using Design Expert Software, where all three factors, including sonication time and temperature, as well as the solvent–solid ratio, had a considerable effect on ASG extraction efficiency (*p* < 0.05). Figure 1 shows the synchronized effect of sonication time and temperature on the yield amount of ASG extracted by 60% ethanol. According to the results, the time has a significant impact on extraction yield, so that with increasing the time from 30 to 45 min, the extraction yield went upward, and the maximum value was obtained in 45 min, but with increasing time to 60 min, the yield again decreased, which relates to the fact that the quadratic effect can be seen in the diagram. The extraction time was substantially impacted, as seen by the three-dimensional (3D) surface plots (Figure 1). The extraction yield of ASG extract improved with the rise of extraction time and the extraction temperature influence, taking into consideration that the longer heating period resulted in a lower extraction yield.

Responses were optimized at the same time, and the model was used to find the best conditions. The optimal conditions for ASG extraction by UAE suggested by the model to gain high yields were specified as follows: a 45 min extraction time, a 40 °C temperature, and a 1:25 g/mL solid–liquid ratio to reach the optimum yield of 75.88 ± 0.94%. These settings were chosen to maximize the yield, with D = 0.9 having the greatest overall desirability. Figure 1 depicts the effects of the experimental levels of tested variables on the response. The contour plots came in a variety of forms, indicating various interactions between factors. Figure 1A shows the effect of extraction time and temperature and their influence on the percentage of extraction yield. The yield of ASG extract improved when the extraction time increased. The ultrasound waves require a certain time to stimulate the cell–wall interference and then release the extract. A similar effect of extraction temperature on the yield of ASG extract was observed. Results showed that when extraction temperature increased, the solubility of ASG also increased, which also improves the extraction yield. The yield of ASG extract also increased because of other reasons like higher solvation, increasing material porosity, and mass transfer, as confirmed by Chan et al. [28]. The impact of extraction temperature on ASG yield and its phytochemical content confirmed by a previous study conducted by Elnour et al. [29] showed that increases in extraction yield in the content of phenolic components of samples resulted from increased extraction temperature. However, the results were that, with increasing temperature from 30 to 40 °C, the extraction yield increased, and there were increases in yield with increasing sonication temperature due to the mass transfer produced by the increase in ASG solubility and the decrease in solvent viscosity. Other research also detailed the important effect of temperature during extraction on the mass transfer of water-soluble polysaccharides, and the increase in extraction efficiency of polysaccharides in the extract while during increasing the temperature up to 60 °C, where the quantity of extracted ASG decreased (Figure 1).

The extraction yield was maximal at a temperature of 40.83 °C and decreased at further increases of temperature. However, the over increase in the temperature led to a decrease in the extraction yield since the extraction temperature exceeded the optimum temperature, as in the case of over 60 °C, due to oxidative degradation and the decrease in solvent ability to dissolve the bioactive compounds, where more than 50% (vol) of the solvent was evaporated. In fact, the present findings are analogous to the results reported by Bi et al. [30]. For this reason, a milder heating condition is considered appropriate for the extraction, where the optimum condition was taken at 40 °C.

Figure 1B displays the response surface plot of extraction time and solid–liquid ratio and their interactions on the percentage of the extraction yield. Extraction time is important parameter in solid–liquid extraction because it affects the solubility and mass transfer of bioactive compounds. Furthermore, as shown in Figure 1B, the yield raised with an increase in extraction time up to 45 min, and then decreased slightly. Prolonged extraction time increases the possibility of oxidation, epimerization, and degradation of bioactive compounds. These results explain the critical role of extraction time in minimizing extraction process costs.

Similar interactions of the extraction temperature and solid–liquid ratio were observed in Figure 1C in the UAE of ASG. Results showed that when extraction temperature increased, the solubility of ASG also increased then decreased. Moreover, the yield was not significantly affected when the sample-to-solvent ratio increased from 1:15 to 1:25 g/mL; therefore, it does not affect minimizing or maximizing the yield response. The solid–liquid ratio’s effect on optimization was studied to increase the extraction efficiency, as well as decreasing production cost and solvent usage. A solid–liquid ratio of 1:25 g/mL was found to give the best results to achieve the optimum extraction yield of 75.87%. To optimize ASG extraction yield, the model equation was utilized to forecast the best extraction conditions. The model’s recommended optimal conditions to gain high yields were specified as follows: a temperature of 40.83 °C with a solid–liquid ratio of 1:25 g/mL and a 45.56 min extraction time. To compare the predicted result with the measured values, triple experiments (*n* = 3) were conducted under optimal circumstances to compare the anticipated result with the measured values.

### 3.4. Validation of the Optimized Parameter

Additional extractions were carried out to validate the generated model and to confirm the best results. The optimized condition was obtained at a temperature of 40 °C, a solid–liquid ratio of 1:25 g/mL, and an extraction time of 45 min. The experimental values were compared with predicted values based on CV% to determine the validity of the model.

Validation tests revealed that the average yield was 74.89 ± 0.32%. These values are close to the predicted yield of 75.87%, indicating that the model is suitable for optimization. The greatest extraction yield of ASG was obtained in UAE extraction with a lower ratio of solvent, greater time, and an appropriate temperature. This study approved the results of another study [31], which confirmed that the yield increased once the time for extraction increased; however, this was under a controlled temperature. The fact that these findings were so closely related proved that the response model was accurate in representing the predicted optimization. It also implies that when/by utilizing UAE, the models can accurately estimate ASG extraction yield. The desirability function was also used to predict one set of optimum conditions for three response variables.

### 3.5. Raman Spectroscopy

**Figure 2:**
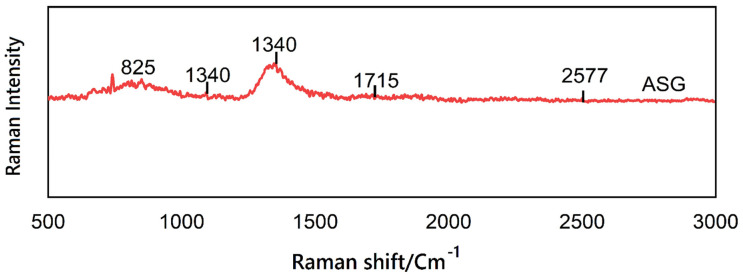
Typical Raman spectrum of *Acacia Seyal* gum powder sample.

The ASG Raman spectrum for ethanol extract is nearly the same compared to the earlier raw gum spectrum. TheRaman spectra of ASG is presented in Table 5. The optimized extracts fingerprint spectra were obtained with the Raman spectrum, in which the ethanol gum ASG sample had three primary peaks of 1330 and 825 cm^−1^. The ASG spectra indicated bands at around 1715 cm^−1^. However, the spectrum ASG indicated two bands of 2905 and 2577 cm^−1^ of diameter (Figure 2). In this research, the spectra of the extreme ribbons were about 2700 cm^−1^, which was ascribed to the N-H and C-H for amin and alkyl, respectively, where the results coincide with Freire et al. [32]. The sharp band is considered a hallmark frequency for the SH and SS groups at 2577 cm^−1^ [33]. At 1333 cm^−1^ of total waves, 1 Raman galactose and glucose band was visible, respectively, as ASG consists primarily of monosaccharide arabinose and galactose units. De Pinto et al. [34] previously reported the same findings in previous investigations and found that acacia gums comprise D-galactose, L-rhamnose, L-parabiotic acids, and two uronic acids, glucuronic acids, and 4-O-methyl-glucuronic acids. The vibration of deformation of C-C-H, C-H_2_ was related to the band seen at 1340 cm^−1^ [35]. The ring deformation was allocated to the sharp band at 1373 cm^−1^. At 1032 and 1083 cm^−1^, the peak was predominantly attributed to the bending vibration of CN bonds in proteins and amino acids, and the vibration of C-H and C-O-H in carbohydrates contributed little. The 800–900 cm^−1^ portion of the reference values for Raman originated in the stretching of a functional C-C skeleton (n-alkane) group, which is a hydrocarbon composed solely of C-C individual bonds and has a C-O-C, C-O-H glycosidic ring stretching sugars [36,37]. In the meantime, sugar bands range between 800 and 1100 cm^−1^ as well as at 1400 cm^−1^ [38]. In addition, the 800 cm^−1^ bands were allocated to the C-H, C-O-H, and CH_2_ curves [39]. A combination of the characteristics of CH_2_ and COO group vibrations formed the absorption bands at 1500 cm^−1^, 1476 cm^−1^, and 1260 cm^−1^, and a single spectral region was attributed to flavanol and organic acid. The results were agreed with by [40,41], who described the quick detection of antioxidant phenolic chemicals.

In addition, Raman spectroscopy has been used to identify carotenoids and indolic alkaloids. Thus, the antioxidant activity of the crude extracts could therefore have revealed a potential function for bioactive substances. Thus, Raman bands at 1301 cm^−1^ assigned to CH_2_, which is assigned to carotenoids, and the bands at 1517 and 1000 cm^−1^ are typical bands for β-carotene [42]. At the region between 1600 and 1800 cm^−^^1^, Raman bands would expect the C=O Alkyl ketone functional group, and a small contribution by the vibration of the C=C groups is expected [43]. The approximate assignments of OH and C-C aliphatic chain vibrations were found at 1155 cm^−1^ and 1266 cm^−^^1^, respectively [44]. A peak for NO2 symmetric stretching was detected at 1290 cm^−^^1^, whereas a peak for C-O stretching was seen at 1326 cm^−1^, while the region between 1400 and 1500 cm^−^^1^ displays CH_2_ and CH_3_ twists. Raman spectroscopy of phenolic antioxidants compound identification provides a systematic assessment of p-coumaric acid, sinapic acid, and caffeic acid (Table 4). Hence, Raman is considered as an effective tool for bioactive compounds discovery [45].

Polysaccharide vibrational bands characterized the Raman spectra of ASG. Weak and poorly resolved signals in wavenumbers above 1600 cm^−1^ explain the low-protein fractions. The results indicate that Raman spectroscopy may be a powerful technique to analyze the natural organic binding medium and identify function groups to effectively evaluate the antioxidant activity and health advantages of specific bioactive compounds in the pharmaceutical sectors.

### 3.6. FTIR Analysis

**Figure 3:**
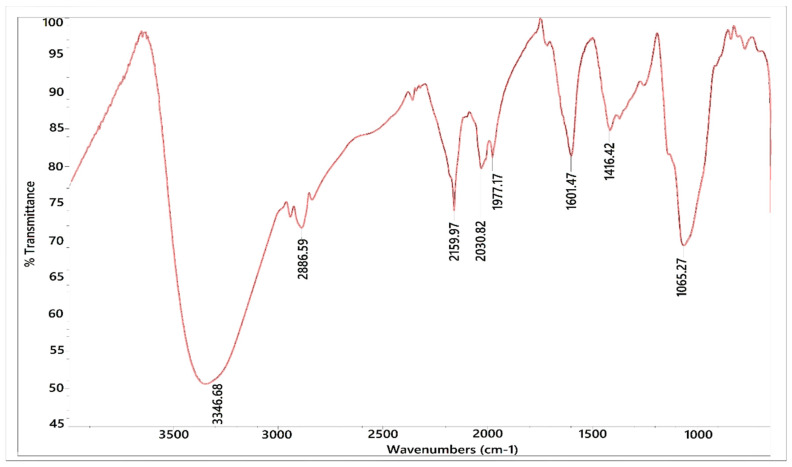
FTIR spectra in the region of 400–4000 cm^−^^1^ *Acacia Seyal* gum powder sample.

FTIR spectra were acquired for ASG extracts between 4000 and 400 cm^−1^. The resulting spectra revealed some substantial overlap of every absorption spectrum of the various components, in which each band presents the characteristic absorption peaks for functional groups obtained from the samples (Table 6). Based on Figure 3, the resulting FTIR spectra and the ASG ethanol crude extract spectra can have an excellent description. The FTIR spectrum of ASG samples had important peaks at approximately the same range of 3642, 2960, 2926, 2888, 2386, 2311, 2087, 2165, 1605, 1503, 1229, 1301, 1191, 1065, and 824 cm^−1^ (Figure 3). In the wavenumber’s region, ASG showed three leading spectral bands connecting 1700 to 1200 cm^−1^. The band allocation may be partially related to the contribution of proteins in ASG. The different band at around 1602 cm^−1^ also presented characteristic bands of C‚C stretch, amide NH bend, and NO2 from both aliphatic and aromatic galactoproteins and amino acids. Amide I and amide II vibration bands are assumed to be nearly 1690 and 1480 cm^−1^ based on the literature for proteins in the FTIR spectra [46]. The groups are supposed to be primarily dependent on the polypeptide backbone secondary structure and not often affected by the characteristics of the side chains [47,48]. Therefore, gum Arabic has distinctive polyphenolics bands with different vibration occurrences. A clear stretch of C-C stretch, amide NH bend, and NO_2_ from both aliphatic and aromatic galactoproteins and amino acids can be seen at approximately 1615 cm^−1^ [49]. Saturated Aliphatic (alkene/alkyl) CH_3_ bend was stated in a wavenumber region at 2960–2880 cm^−1^ [50]. At a medium band in the region of 1250–1020 cm^−1^, amine C-N stretch was observed [51].

At the area of 3285–2600 cm^−1^, the FTIR spectrum showed a broad OH stretching vibration in carboxylic acids, while the same area of the stretching vibration region for carbon and aromatic C-H groups also showed FTIR spectra of specific polysaccharides [52]. However, the band position near 1229 cm^−1^ was found to depend on the biochemical composition and specifically the phenol content [53]. Therefore, additional studies are needed for identifying the specific functional groups in gum Arabic. The raw ASG contains glucuronic acid content in a considerable amount and a low content of proteins. As a result, the assignment of the absorbance bands at 1608 cm^−1^ suggests a carbonyl C-O that could be because of glucuronic acid in the ASG spectrum [54]. Moreover, IR bands at 1301 cm^−1^ with the bending of secondary aromatic amines showed the contribution of proteins’ imines [55]. This segment, also known as the carbohydrate fingerprint region, is created by the side chains of polysaccharides and skeletal stretching vibrations. Even though the band at 2159 cm^−^^1^ represents the ethene CH antisymmetric stretch compound and CH_2_=CH_2_ as a functional group, the bands at 2926 cm^−1^ indicate the presence of the most frequent plant gum polysaccharides like sugars, galactose, arabinose, and rhamnose as well as the presence of alkane, and these results agreed with Daoub et al. [55], who performed an FTIR study of Acacia gum. The maximum ASG absorbance peaks found at 1747 cm^−1^ somewhere in the infrared spectral bands between 1200 and 824 cm^−1^ are difficult for interpretation and were thought to relate to the carbohydrate group of Acacia gums. The skeletal stretching vibrations and the polysaccharides’ side chains create this so-called carbohydrate fingerprint area, which is controlled by ring vibration with extending vibrations. They are consistent with the FTIR study on raw *Acacia Senegal* and ASG carried out by Lopez-Torrez et al. [56]. The results showed the existence of alcohol, alkanes, aromatics, imines or ketones, alkenes, phenols, and amines functional groups.

Based on the above interpretation, the spectroscopic description of the ASG and its elements (phenolics and polysaccharides) by FTIR showed the presence of aromatic galactoproteins, and amino acids with sugars, galactose, arabinose, and rhamnose. These distinct peaks may offer a simple, quick, and low-cost method of identification. However, these findings provide a solid foundation for follow-up research on ASG.

### 3.7. Chemical Profile of Ethanol ASG Extract Using GC-TOFMS

The GC-TOFMS analysis used for the identification of chemical constituents of the ethanol extract of ASG was performed using a time-of-flight mass spectrometry GC-TOFMS apparatus, which is frequently employed in the field of cultural heritage to identify the sugar content of polysaccharide materials [57]. These chemicals identified by GC-TOFMS analysis were compared to compounds found in earlier Arabic gum studies. More detailed analysis was done; however, some of their activities were identified and reported and others were not. The chemical profile of ASG was determined by comparing the similarity of the pattern resulting from the GC-TOFMS reading with the pattern of the available database, which revealed the presence of several chemical constituents that have been reported to have many therapeutic values and biological properties. This list of compounds identified by GC-TOFMS analysis was compared with compounds in the library of ASG extract phytochemical analysis. More detailed analysis was done for those compounds, and their activities were identified and reported. The constituents of ASG generally belong to the class of polysaccharide, alkaloids, flavonoids, phenols, and tannins. According to many previous studies, these groups of compounds have potential activity as antidiabetic, antioxidant, and anticancer compounds. The result of the GC-TOFMS analysis of the ethanolic extract ASG is shown in Table 7 where the interpretation of mass spectrum of GC-MS was done using the database of the National Institute Standard and Technology (NIST), and the mass spectrum of the unknown component was compared with the spectrum of the known component stored in the NIST library. The compound name, probability, molecular formula, molecular weight, and biological activity of the test materials were determined.

A GC-TOFMS analysis in this study of an ethanolic extract of ASG revealed the presence of different phytochemicals that may contribute to the plant’s therapeutic bioactivity. The GC-TOFMS spectrum approved the presence of different components with different retention times as illustrated in Table 7. However, the observed mass spectra consider the fingerprint of these compound that can be identified from the data library. It was widely reported that Arabic gum contained a considerable number of phytochemicals that exhibit antioxidant activity and can combat many diseases such as renal, cardiovascular, gastrointestinal, and respiratory diseases, due to its agonist effect on the oxidative stress and DNA damage [92]. Furthermore, Badreldin et al. verified the antioxidative and anti-inflammatory properties of *AG*. It was found that amino-saccharins like 1,2-Benzisothiazol-3-amine which is one of tert-butyldimethylsilyl (TBDMS) derivatives which are considered to be antioxidant compounds, were investigated previously in the methanol extract of *Dillenia scabrella.* The 1,2,3,12a-Tetrahydro-6,9,10-trimethoxy-2-oxo-12H-benz(6,7) oxepino(2,3,4-i,j) isoquinoline alkaloid compound with antitumor activity was also detected and reported on by various researchers for its biological activities [93]. R)-(-)-2-Amino-1-propanol (alaninol) is one of the amino alcohol derivatives that poses antioxidant and anti-proliferative activity [94]. Compound like Monoalkylamines, isobutylamine, and N, N-dimethyl-4-nitroso-3-(trimethylsilyl) aniline, are phenylamine compounds with antitumor activity found in the analysis results [95]. 9,12,15-Octadecatrienoic acid, 2,3-bis [(trimethylsilyl) oxy] propyl ester, (Z, Z, Z) are also anti-inflammatory, hypocholesterolemia, and cancer-preventive compounds [96,97]. Astaxanthin, β-carotene, Phenylindolizine, and similar types of compounds were identified in methanol extract of ASG [98]. Although the previous literature survey confirmed the occurrence of 2,4-Dimethoxycinnamic acid [98] and cyclobarbital from various plant sources [99,100], there is no report of GC-TOFMS-based metabolite profiling to detect the unique presence of benzodioxole and barbital derivatives from any part of ASG. Cyclobarbital, also shown in the results, which is used therapeutically in the treatment of insomnia and as an anesthetic, anti-convulsant, and skeletal or muscle relaxant in recent times, has also been also reported to have anti-proliferative potential [62]. Other compounds were also present, like Cyclotrisiloxane and hexamethyl- which had broad-spectrum antimicrobial activity, so we concluded that ethanol extracts of ASG can be a good source of antioxidant and antimicrobial compounds [69,70] discovered that Spirilloxanthin, Rhodoviolascin, β-carotene, lycopene, and astaxanthin are carotenoids that play an important role as antioxidants; besides that, they have potential therapeutic significance in pain and inflammation management [101,102]. Another compound was reported Deserpidine, which is a naturally occurring alkaloid that has been used for the treatment of hypertension and as a tranquilizer. In addition, it appears to act as a controller of other cardiac disorders [84]. These identified compounds were observed to be present in the extract of different plant parts and exhibiting different biological activity, for example anticancer activity.

## 4. Conclusions

The green extraction technique (UAE) can be a potential alternative for conventional extraction methods since it improves the yield and quality of the extract. The RSM generated optimum extraction conditions as follows: 40 °C of extraction temperature, 45 min of extraction time, and 1:25 g/mL of solid–liquid ratio. This could be utilized in future ASG extractions on a larger scale by considering these parameters for economic evaluation. Moreover, the outcomes from the RAMAN, FTIR, and GC-TOFMS profile suggest that ASG gum phytochemical compounds could be a potent source for active natural compounds like glycosides, alkaloid, triterpenes, flavonoids, quinones, coumarins, and polyphenolic compounds that could be used to develop novel therapeutic agents for a variety of diseases. They might also be a possible reason to exhibit anticancer, antioxidant, and antibacterial activities. This study could be useful to lead the path for future developments of pharmaceutical applications of ASG.

## Figures and Tables

**Table 1:** Experimental design and levels of independent process variables.

Symbol	Independent Variables	Low Level	High Level
A	Extraction Time (min)	30	60
B	Extraction Temp (°C)	30	50
C	Solid–Liquid Ratio (g/mL)	15	25

**Table 2:** FCCCD experimental design and the response.

Extraction Condition Yield (%)					
Run	A: Extraction Time (min)	B: Extraction Temp (°C)	C: Solid–Liquid Ratio (g/mL)	Actual	Predicted
1	30	30	25	65 ± 0.02	65.28
2	30	40	20	70 ± 0.01	69.14
3	45	30	20	70 ± 0.03	70.8
4	30	30	15	65 ± 0.27	64.60
5	60	30	15	64 ± 0.15	63.54
6	60	30	25	65.3 ± 0.1	64.89
7	45	40	20	76 ± 0.08	75.39
8	60	50	15	66 ± 0.06	65.66
9	45	40	20	76 ± 0.41	75.39
10	45	40	15	74 ± 0.09	74.60
11	30	50	25	65 ± 0.09	65.39
12	60	50	25	67 ± 0.54	67.34
13	45	40	20	75 ± 0.08	75.40
14	45	40	20	76.3 ± 0.01	75.40
15	45	40	25	76.6 ± 0.05	75.97
16	45	40	20	74 ± 0.08	75.39
17	45	50	20	73 ± 0.51	71.92
18	45	40	20	75 ± 0.53	74.60
19	30	50	15	64 ± 0.12	64.30
20	60	40	20	69 ± 0.2	69.60

**Table 3:** ANOVA for a fitted quadratic model of extraction conditions with yield (%).

Source	Sum of Squares	Degree of Freedom	Mean Square	F-Value	*p*-Value	
Model	431.23	9	47.91	53.2	<0.0001	significant
A-Time	0.54	1	0.54	0.6	0.0045	
B-Temp	3.21	1	3.21	3.57	0.0088	
C-S/L	3.59	1	3.59	3.98	0.07	
AB	2.73	1	2.73	3.03	0.11	
AC	0.22	1	0.22	0.25	0.63	
BC	0.06	1	0.06	0.06	0.81	
A^2^	99.96	1	99.96	111	<0.0001	
B^2^	44.64	1	44.64	49.57	<0.0001	
C^2^	0.11	1	0.11	0.12	0.74	
Residual	9.01	10	0.9006			
Lack of Fit	5.14	5	1.03	1.33	0.38	not significant
Pure Error	3.86	5	0.77			
Cor Total	440.24	19				

**Table 4:** Confidence and prediction interval ANOVA analysis results.

Response	Predicted Mean	Predicted Median	Observed	Std Dev	SE. Mean	95%CI Low for Mean	95%CI High for Mean	95%TI Low for 99% Pop	95%TI High for 99% Pop
yield	75.88	75.88	74.89	0.94	0.66	74.39	77.35	70.78	80.97

**Table 5:** Functional groups assigned peaks of *Acacia Seyal* gum using Raman spectroscopy.

Wave Number cm^−1^	Approximate Assignment	Functional Group
3200	O-H	phenols
>2700	N-H, and C-H stretching modes	Amin, Alkyl
2577	S-H, S-S	Sulfhydryl
1500–1700	C=O and C=N stretch	Alkyl ketone
1500–1550	N-O stretching	Nitro compound
900–1200	C-C stretching, CH_3_, CH_2_, C=O	Alkane
1461	C-H bending	Methylene group
1340	C-C-H, C-H_2_ bending	Alkane.
1333	CH_2_ vibrations	Monosaccharide galactose, glucos
1326,1261	C-O stretching	Alkyl Ester
1301	CH_2_	Carotenoids
1078	C-O, C-C, C-OH	Carbohydrate, Monosaccharides
941, 979	C-C skeletal stretch, C-O-C	alkane, glycosidic linkage
600–650	O-C=O	Acetate Ester

**Table 6:** Main functional groups assigned to the different vibrations present in FTIR spectra.

Possible Band Assignment	Wavenumber (cm^−^^1^)	Functional Group
O-H···O	3347	Alcohol and hydroxy compound [50]
C-H or/and NH_3_	2888	amino acids [52]
OH stretch	3642	Primary alcohol
C-H	2960	alkene/alkyl
CH_2_=CH_2_	2927	Alkene
C-H stretching	2087	Aldehyde
C=O	1731–1713	ketones
	1730–1705	Ketone [54]
C=C	1624	Alkene [53]
Phenolic OH	1229	
C-N	1301	Secondary amides [53]
C-O-C	1140	Ether [56]
CN stretch	1065	Primary amine
C-H aromatic	824	alkene

**Table 7:** List of compounds of ASG ethanolic extracts observed in GC-TOFMS with their retention time and biological activity.

Compound Name	RT (min)	M.F	M.W (g/mol)	Biological Activity	Reference.
Lupulon	14.28	C_26_H_38_O_4_	414	antimicrobial	[58]
2-Butynedioic acid Acetylenedicarboxylic acid	59.719	C_4_H_2_O_4_	114	antibacterial potency	[59]
7-Methyl-Z-tetradecen-1-ol acetate	29.71	C_17_H_32_O_2_	268	anti-inflammatory	[60]
9-Octadecenal	19.52.78	C_18_H_34_	266	antibacterial Membrane stabilizer	[61]
Benzene, 1,3,5-trimethyl-2-octadecyl	51	C_27_H_48_	372	not reported	
Cyclobarbital	55.4	C_12_H_16_N_2_O_3_	23	anti-proliferative	[62]
Rhodoviolascin	57	C_42_H_60_O_2_	597	bacterial metabolite. antioxidant	[63]
β-Carotene	56	C_40_H_56_	537	antioxidant	[64]
2,4-Dimethoxycinnamic acid	2	C_11_H_12_O_4_	208	cytotoxic activity	[65]
Benzoic acid 2-methylpentyl ester	61	C_13_H_18_O_2_	206	antimicrobial	[66]
2-Phenylindolizine	64	C_14_H_11_N	193	antimicrobial activity	[67]
Cyclotrisiloxane, hexamethyl	64	C_6_H_18_O_3_Si_3_	222	antimicrobial antibacterial and antioxidant activity	[68,69,70]
Chlortetracycline	58	C_22_H_24_Cl_2_N_2_O_8_	515	antibacterial agents	[71]
Astaxanthin	58	C_40_H_52_O_4_	596	antioxidant anticancer	[72]
Acridine, 9-methyl-	64	C_14_H_11_N	193	not reported	
Isopilocarpine	63	C_11_H_16_N_2_O_2_	208	antimicrobial	[73]
Betamethasone	56	C_22_H_29_FO_5_	392	anti-inflammatory	[74]
Benzo[h]quinoline,2,4 dimethyl-	56	C_15_H_13_N	207	anticancer	[75]
Benzamide, N-ethyl-N-(3-methylphenyl)-4-ethyl-	57	C_17_H_19_NO	253	antibacterial	[76]
Pyrrolidine, 1-(1-oxo-5,8-octadecadienyl)	59.62	C_18_H_31_NO	277	anti-inflammatory and antitumor activity	[77]
5H-Cyclohepta[b]pyridine-3-carbonitrile,6,7,8,9-tetrahydro-2-amino-4-(2-fluorophenyl)-	61.85	C_11_H_12_N_2_	172	not reported	
Lycopene	63.84	C_40_H_56_	69	antioxidant	[78]
Trisiloxane, 1,1,1,5,5,5-hexamethyl-3,3-bis[(trimethylsilyl)oxy]-	56.47	C_12_H_36_O_4_Si_5_	384	antioxidant	[79]
1,3-Dioxolane,2-(1 phenylethyl)	62.24	C_11_H_14_O_2_	178	not reported	
D-Glucopyranosiduronic acid, 3-(5-ethylhexahydro-1,3-dimethyl-2,4,6-trioxo-5-pyrimidinyl)-1-methylbutyl 2,3,4-tris-O-(trimethylsilyl)	59.1	C_29_H_56_N_2_O_10_Si_3_	677	antibacterial and antioxidant	[80]
Oxazolam	60.85	C_18_H_17_ClN_2_O_2_	328	muscle relaxants	[81]
Cyclohexyldimethylsilyloxy-3-phenylpropane	50	C_15_H_22_O_2_	330	anticancer and antitumor activities	[82,83,84]
Glycine,N-[(3à,5á,7à,12à)-24-oxo-3,7,12 tris[(trimethylsilyl)oxy]cholan-24-yl]-,methyl ester	10	C_32_H_38_N_2_O_8_	578	antihypertensive action	[85]
1,2-Benzisothiazol-3-amine tbdms	4	C_13_H_20_N_2_SSi	264	antifungal activity	[86]
R)-(-)-2-Amino-1-propanol	12	C_3_H_9_NO	75	antioxidant and antiproliferative activity	[87]
Silicic acid, diethyl bis(trimethylsilyl) ester	57	C_10_H_28_O_4_Si	296	antibacterial antioxidant activity	[88,89]
6,9,10-Trimethoxy-12H-benz (6,7) oxepino(2,3,4-i,j)isoquinoline	23	C_19_H_17_NO_4_	323	antitumor	[90]
N, N-dimethyl-4-nitroso-3-(trimethylsilyl) aniline	30	C_11_H_18_NOSi_2_	222	antitumor activity	[91]

## Data Availability

The data presented in this study are available within the article.
